# Pathways to polyploidy: indications of a female triploid bridge in the alpine species *Ranunculus kuepferi* (Ranunculaceae)

**DOI:** 10.1007/s00606-017-1435-6

**Published:** 2017-07-06

**Authors:** Christoph C. F. Schinkel, Bernhard Kirchheimer, Stefan Dullinger, Danny Geelen, Nico De Storme, Elvira Hörandl

**Affiliations:** 1 Department of Systematics, Biodiversity and Evolution of Plants (with Herbarium), University of Goettingen, Untere Karspüle 2, 37073 Göttingen, Germany; 2 Department of Botany and Biodiversity Research, University of Vienna, Rennweg 14, 1030 Vienna, Austria; 3 Department of Plant Production, Faculty of Bioscience Engineering, Ghent University, Coupure Links 653, 9000 Ghent, Belgium

**Keywords:** Apomixis, Flow cytometric seed screen, Pollen size, Polyploidy, *Ranunculus kuepferi*, Triploid bridge

## Abstract

Polyploidy is one of the most important evolutionary processes in plants. In natural populations, polyploids usually emerge from unreduced gametes which either fuse with reduced ones, resulting in triploid offspring (triploid bridge), or with other unreduced gametes, resulting in tetraploid embryos. The frequencies of these two pathways, and male versus female gamete contributions, however, are largely unexplored. *Ranunculus kuepferi* occurs with diploid, triploid and autotetraploid cytotypes in the Alps, whereby diploids are mostly sexual, while tetraploids are facultative apomicts. To test for the occurrence of polyploidization events by triploid bridge, we investigated 551 plants of natural populations via flow cytometric seed screening. We assessed ploidy shifts in the embryo to reconstruct female versus male gamete contributions to polyploid embryo and/or endosperm formation. Seed formation via unreduced egg cells (B_III_ hybrids) occurred in all three cytotypes, while only in one case both gametes were unreduced. Polyploids further formed seeds with reduced, unfertilized egg cells (polyhaploids and aneuploids). Pollen was highly variable in diameter, but only pollen >27 μm was viable, whereby diploids produced higher proportions of well-developed pollen. Pollen size was not informative for the formation of unreduced pollen. These results suggest that a female triploid bridge via unreduced egg cells is the major pathway toward polyploidization in *R. kuepferi*, maybe as a consequence of constraints of endosperm development. Triploids resulting from unreduced male gametes were not observed, which explains the lack of obligate sexual tetraploid individuals and populations. Unreduced egg cell formation in diploids represents the first step toward apomixis.

**Electronic supplementary material:**

The online version of this article (doi:10.1007/s00606-017-1435-6) contains supplementary material, which is available to authorized users.

## Introduction

Polyploidization is generally defined as the acquisition of more than the two nuclear chromosome sets found in diploids. Discovered more than a century ago (Strasburger 1910) and naturally occurring in many eukaryotic taxa (Wood et al. 2009) polyploidy is nowadays recognized as an evident and important force of evolution (Ramsey and Schemske 1998), especially in plants (Karpechenko 1927; Stebbins 1950, 1971) and particularly in angiosperms (Soltis and Soltis 2000; Wendel 2000). Fossil records indicate that up to 70% of angiosperm plants are of polyploid origin (Masterson 1994). More recent genomic analyses even suggest higher proportions of ancient polyploidization events in species’ histories to the point that all seed plants are estimated to be paleopolyploid (Blanc et al. 2003; Jiao et al. 2011). This further indicates the importance for whole genome doubling (WGD) as driver for adaptation and speciation (Levin 1983; Soltis and Soltis 2009; Wood et al. 2009).

Besides many possible associated costs, the fundamental life history changes frequently accompanying the genomic excess of polyploidy, such as phenotypic and/or physiological plasticity, can convey polyploids an evolutionary advantage over their diploid progenitors (Hegarty and Hiscock 2008). Rapid range expansions of recently formed polyploids (Levin 1983; Hull-Sanders et al. 2009; Treier et al. 2009) and distinctly larger distribution areas of apomictic polyploids (van Dijk 2003; Kearney 2005; Hörandl 2006; Hörandl et al. 2008; Randle et al. 2009) which mostly extend to higher and accordingly cooler latitudes and elevations (Bell 1982; Bierzychudek 1985; Schinkel et al. 2016), are the most intriguing indications of such benefits.

However, despite the seemingly striking evolutionary importance of polyploidy, even the most crucial steps of polyploid evolution, their formation and establishment, are not fully understood. Although genetic events leading to polyploidy can occur frequently (Soltis and Soltis 1999; Soltis et al. 2004), neopolyploids have to overcome certain barriers to form viable populations and to persist (Levin 1975). Baack et al. (2005) pointed out that polyploidization must take place within diploid progenitor populations (sympatric), leaving them prone to pollen flow from diploids. The resulting triploids are assumed of lower fitness. According to the minor cytotype exclusion theory, neotetraploids would thus be quickly eliminated from the population (Levin 1975; Husband 2000). In this context, one major side effect of polyploidization is the immediate establishment of strong post-zygotic barriers, a mechanism leading to reproductive isolation (Ramsey and Schemske 1998, 2002). Alternatively, or in addition, polyploids may avoid genetic interaction with diploids by pre-zygotic barriers such as a switch to selfing or asexual modes of reproduction (Levin 1975), a phenological separation from their diploid ancestors through changes in flowering time (Segraves and Thompson 1999), or an ecological differentiation, i.e., by shifting the optima and/or expanding the breadths of their ecological niches (spatial separation) (Husband and Schemske 2000; Levin 2004). However, recent studies did not support niche divergence as an essential prerequisite for successful polyploid establishment (see Glennon et al. 2014, Kirchheimer et al. 2016).

Natural polyploid formation can occur through different pathways. Sexual polyploidization is usually based upon meiotic aberrations resulting in unreduced gametes, with pollen and/or egg cells exhibiting a somatic chromosome set (Bretagnolle and Thompson 1995; Ramsey and Schemske 1998; Brownfield and Köhler 2011; De Storme and Geelen 2013). Meiotic restitution is, similar to mitosis, a non-reductional cell division process in which dyads (and occasionally triads)-containing spores with the somatic chromosome number are produced instead of the tetrads-containing spores with the gametophytic chromosome number that are normally formed through regular meiotic cell division. Meiotic non-reduction has been recognized in numerous plant taxa and seems to be the predominant mechanism of diploid gamete formation (Bretagnolle and Thompson 1995; Bicknell and Koltunow 2004; Pecrix et al. 2011; De Storme et al. 2012; De Storme and Geelen 2013; Lovell et al. 2013; Mirzaghaderi and Hörandl 2016).

Meiotic restitution during female sporogenesis results in two unreduced megaspores, from which one develops into an unreduced embryo sac containing an unreduced egg cell and polar nuclei (diplospory). Female meiosis, however, can be also bypassed and an unreduced embryo sac can also develop from a somatic cell of the nucellus (apospory). These two mechanisms are components of gametophytic apomixis (Koltunow and Grossniklaus 2003). Nevertheless, asexual production of gametes is not exclusive and mostly accompanied by sexual gamete formation in parallel (Aliyu et al. 2010; Cosendai et al. 2013; Dobeš et al. 2013; Hojsgaard et al. 2013, 2014; Klatt et al. 2016). Simultaneous formation of unreduced and reduced gametes may result in sporadic merging of both types of gametes, depending on individual frequencies and spatial distribution. In particular, in apomictic species, the fusion of an unreduced egg cell with a reduced pollen grain constitutes a developmental pathway of partial apomixis utilizing a combination of unreduced embryo sac formation (apomeiosis) and subsequent fertilization. Such fertilized apomictic seeds result in the so-called B_III_ hybrids (Nogler 1984; Bicknell et al. 2003), which cause polyploidization and are a potential pathway to fully functional apomixis when coupled to parthenogenesis (Hojsgaard et al. 2014). In contrast, polyploid induction resulting from the fertilization of a reduced egg cell by an unreduced pollen grain does not affect mode of reproduction and just results in sexual polyploidization (Mason and Pires 2015).

Incidence of these so-called 2*n* gametes in natural populations is far more ubiquitous than previously thought. When involved in fertilization events, 2*n* gametes directly produce polyploid offspring and are therefore considered a major pathway to polyploid formation (Bretagnolle and Thompson 1995; Ramsey and Schemske 1998; Otto and Whitton 2000; De Storme and Geelen 2013; Tayalé and Parisod 2013). Although production of unreduced gametes has been observed in many plant taxa, frequencies of unreduced egg cells are highly variable in seeds (Bicknell and Koltunow 2004; Sharbel et al. 2009; Aliyu et al. 2010; Hojsgaard et al. 2014; Klatt et al. 2016), as well as in sperm nuclei in pollen (Rani et al. 2013; Sora et al. 2016). Recent studies demonstrate that environmental stress, especially temperature, influences the production of 2*n* gametes (Lokhande et al. 2003; De Storme et al. 2012; De Storme and Geelen 2013; Mirzaghaderi and Hörandl 2016). But also photoperiod (Quarin et al. 1986; Keller and Körner 2003; Kurepin et al. 2007; Klatt et al. 2016) and drought stress have been reported as factors driving 2*n* gamete formation. These stressors can increase frequencies of unreduced embryo sac or pollen grain production to numbers sufficiently high to explain estimated rates of polyploid formation (10^−5^ for autotetraploids, 10^−4^ for allotetraploids; see Ramsey and Schemske 1998). However, bilateral sexual polyploidization events in which male and female unreduced gametes fuse have the joint probability of two rare events plus the imponderabilities of their temporal and spatial co-occurrence and thus are considered rare in natural populations (Ramsey and Schemske 1998; Husband 2004). Contrary to one-step polyploidization (tetraploid induction), the relatively high incidence of triploids in natural populations of many plant taxa (Dobeš et al. 2004; Husband 2004; Schranz et al. 2005; Voigt et al. 2007) suggests an alternative pathway of autotetraploid formation in two steps, with triploids serving as intermediates (Ramsey and Schemske 1998; Husband 2004). This process, called triploid bridge, involves the fusion of an unreduced with a regularly reduced gamete of diploid parent plants producing triploid offspring. In turn, these triploid intermediates may possibly generate tetraploid offspring through selfing or through crossing with either diploid progenitors or other triploids, depending on their fertility (Husband 2004; Köhler et al. 2010; Mason and Pires 2015).

Triploid bridges as pathway to neotetraploid formation may be hampered by triploid blocks (Comai 2005; Köhler et al. 2010). In some plant species, the parental chromosome dosage in the endosperm seems to be critical for seed development and fertility. Deviations from the usual 2:1 ratio between maternal and paternal chromosome contributions disorganize the expression of parentally imprinted genes necessary for normal endosperm development (Köhler et al. 2010; Lu et al. 2012; Haig 2013). Seeds with vestigial endosperm tend to abort or are mostly infertile (Brink and Cooper 1947). Hence, the sexual fusion of a haploid reduced with a diploid unreduced gamete results in a seed with a triploid embryo but imbalanced endosperm that probably blocks triploid seed formation (Ramsey and Schemske 1998; Spielmann et al. 2003). Moreover, in case triploid seeds do survive, the unequal chromosome number and severe difficulties regarding chromosomal pairing and segregation in meiosis make triploids gametophytically unstable, producing a variety of euploid (1*n*, 2*n*, or 3*n*) and aneuploid gametes (Husband 2004). Lower frequencies of euploid gametes in turn may block tetraploid formation (Ramsey and Schemske 1998). Ramsey and Schemske (1998) evaluated that viable triploids in natural populations produced a surprisingly large amount of fertile euploid gametes. However, this statement refers primarily to pollen, for which a mean fertility of 31.9% is reported in triploid angiosperms. Reliable figures for female gametes are scarce (Bicknell and Koltunow 2004; Parisod and Besnard 2007; Sharbel et al. 2009; Schinkel et al. 2016; Wang et al. 2016) and need to be further investigated.

In this context, our study explores the possible occurrence of triploid bridges and triploid blocks among natural populations of the alpine plant *Ranunculus kuepferi*. The species occurs with diploid, triploid and autotetraploid cytotypes in the Alps, whereby diploids are mostly sexual, while tetraploids are facultative apomicts (Cosendai and Hörandl 2010; Schinkel et al. 2016). Previous studies of the species focused on the distribution of modes of reproduction, but the mode of polyploidization in this species was so far unknown. The observation that triploids occur in the contact zone of diploids and tetraploids prompted us to investigate whether these represent examples of a triploid bridge for polyploidization or just result from secondary backcrosses of established tetraploids with diploids. The amount of unreduced pollen formation in *R. kuepferi* was so far not investigated.

The aim of this study is (1) to analyze ploidy shifts in the seeds of diploid, triploid and tetraploid mother plants via flow cytometric seed screening; (2) to analyze pollen size as putative indicator of unreduced male gamete formation; (3) to evaluate results with respect to the occurrence of a female versus a male triploid bridge-based polyploidization, putatively antagonized by endosperm imbalances acting as a triploid block. Finally, we will discuss implications of our results for the evolution of polyploidy and apomixis.

## Materials and methods

### Plant material

Living plants of *Ranunculus kuepferi* were collected from 81 populations throughout the Alps and transferred to the Botanical Garden of the University of Göttingen as previously described by Schinkel et al. (2016). Our sampling included 18 diploid, 52 tetraploid and 11 populations with mixed cytotypes (2*x*, 3*x*, 4*x* and 5*x*) (Schinkel et al. 2016). The mixed populations occur in the contact zone of diploids and tetraploids. For details on localities and collectors see Schinkel et al. (2016). Voucher specimens have been deposited in the herbarium GOET. In total, 1074 plants have been collected in early fruiting stage. Hence, ovule development, fertilization and seed formation had been completed under natural conditions in buds before collection (Schinkel et al. 2016), which is a prerequisite for analysis of developmental pathways without influences by artificial stress (e.g., cut out, transfer). Due to poor seed set in many individuals, we restricted sampling of seeds for later analysis to 551 plants with a minimum of at least five well-developed seeds. Mature achenes were gathered by bagging fruiting heads in perforated plastic pouches and were kept at room temperature for later analysis.

### Flow cytometry

Determination of the somatic ploidy level of all mother plants was performed by standard methods using 0.5 cm^2^ fresh leaf material per individual (Schinkel et al. 2016). To discriminate between sexual and apomictic pathways in seed development, we used flow cytometric seed screening (Matzk et al. 2000) with minor modifications (Schinkel et al. 2016). Within most facultative apomicts, a single plant is capable of producing both sexual and apomictic seeds even inside the same flower (e.g., Aliyu et al. 2010; Dobeš et al. 2013). Based on this, we realized quantification of reproduction modes by determining the ploidy of both the endosperm and the embryo on a single seed level. Leaf samples of *Zea mays* strain CE-777 provided by J. Doležel were used as external standard and referenced every 96 runs.

For each plant, the ploidy level pattern of five seeds was analyzed following a two-step protocol by Doležel et al. (2007) using Otto I and Otto II buffers, conducted on a CyFlow Space (Partec, Münster, Germany) with FloMax 2.2.0 (Quantum Analysis GmbH, Münster, Germany) operating software. Peak ranges were set manually and calculated as Gaussian means.

Calculated ratios between endosperm and embryo ploidies provided a basis for discrimination between sexual (3:2) and apomictic (3:1, 2.5:1, 2:1) development without ploidy shifts in the embryo. For the respective peak ratios, a threshold of 1.65 was used to distinguish between sexual and apomictic seed. More detailed examinations of plausible developmental pathways followed Matzk et al. (2000), Talent and Dickinson (2007), Cosendai and Hörandl (2010) and Dobeš et al. (2013) and were adjusted accordingly (Schinkel et al. 2016).

**Fig. 1:**
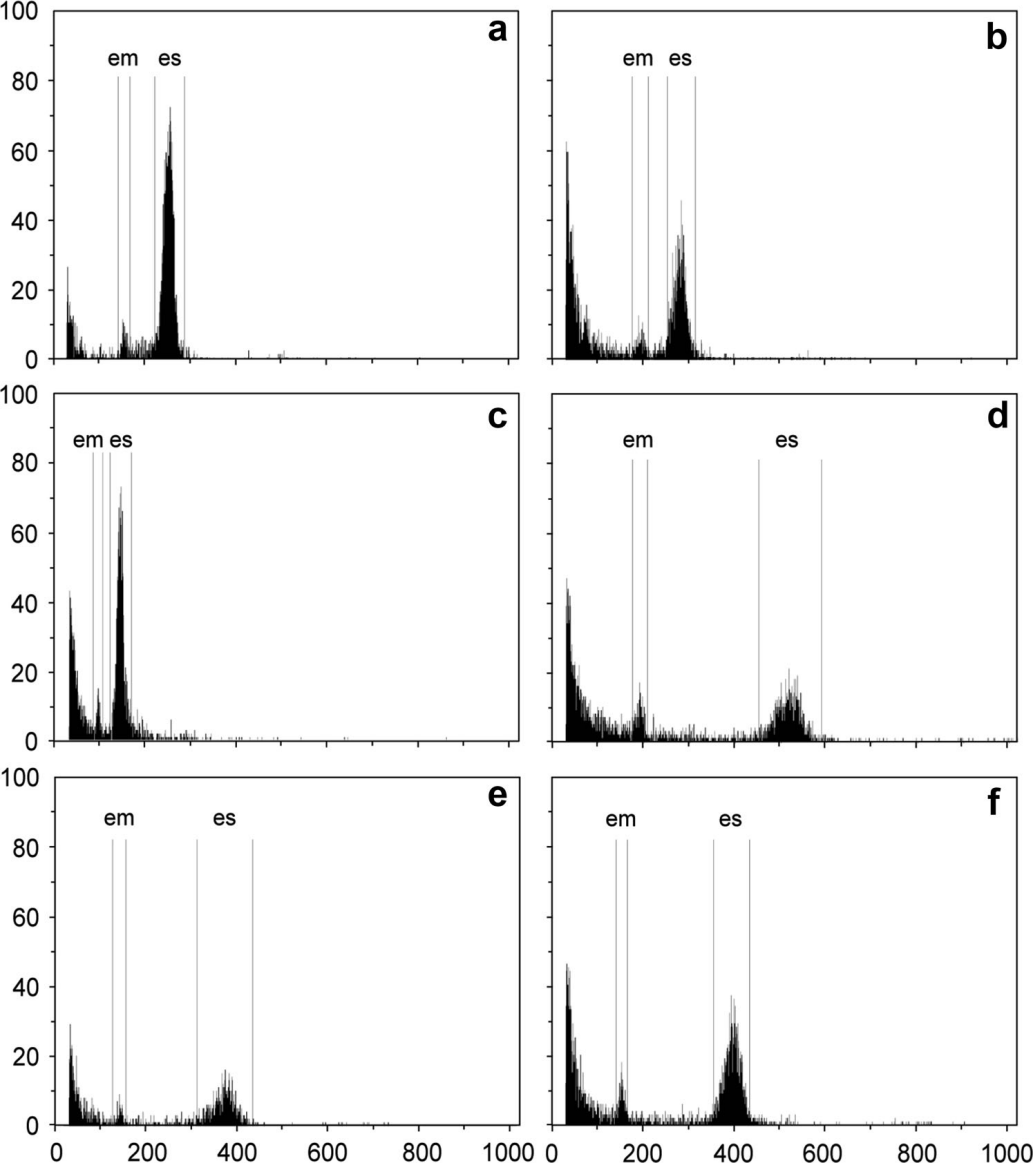
Flow histograms of six seed formation pathways with ploidy shifts in the embryo inferred from FCSS in *Ranunculus kuepferi* (see Table 1). **a** Uniparental polyploidization on a diploid mother plant, triploid embryo, pentaploid endosperm (first step in tetraploidization via female triploid bridge); **b** biparental polyploidization with diploid mother plant, tetraploid embryo, hexaploid endosperm (one-step tetraploidization or 2*n* pollen from tetraploid pollen donor); **c** progeny from triploid mother plant with diploid embryo, triploid endosperm; **d** uniparental polyploidization with triploid mother plant, tetraploid embryo and decaploid endosperm, indicating trinucleate es (second step in tetraploidization via female triploid bridge); **e** polyhaploid progeny from tetraploid mother plant with triploid embryo, hexaploid endosperm (trinucleate es or es fertilization with 2 sperm nuclei); **f** triploid embryo with octaploid endosperm from a tetraploid mother plant, indicating irregular female meiosis; *em* embryo peak; *es* endosperm peak

Here, in this study, we specifically evaluated cases of ploidy shifts in the embryo. Results were pooled for ploidy levels of the mother and developmental pathways (Table 1). Seeds were categorized (after Nogler 1984; Bicknell et al. 2003) either as B_III_ hybrids (unreduced egg cell fertilized by reduced pollen = female triploid bridge; we apply this term to all cytotypes), polyhaploids (reduced egg cell of tetraploid without fertilization), disturbed sexuals (irregular male or female meiosis resulting in aneuploidy), biparental polyploidization (unreduced egg cells fertilized by unreduced pollen) or male triploidization (reduced egg cell fertilized by unreduced pollen). These cases can be discriminated by the respective embryo/endosperm ploidy pattern analysis (when ploidy of the mother plant was known), see flow histograms in Fig. 1 and interpretations in Table 1. Terminology for denotation of ploidy levels after DNA content and especially maternal as well as paternal genome contributions followed Greilhuber et al. (2005).

**Table 1:** Seeds with ploidy shifts in the embryo according to flow cytometric seeds screening (for seeds without ploidy shifts see Schinkel et al. 2016)

Ploidy					Endosperm ratio (maternal/paternal)	Reproduction mode	***N***
Mother plant	Egg cell	Sperm nuclei	Embryo	Endosperm			
Diploid							
2*x*	2*x*	1*x*	3*x*	5*x*	4:1	B_III_	3
2*x*	2*x*	2*x*	4*x*	6*x*	2:1	Biparental polyploidization	1
							(Total 4)
Triploid							
3*x*	1*x*	1*x*	2*x*	3*x*	2:1	Irregular female meiosis	2
3*x*	3*x*	1*x*	4*x*	7*x*	6:1	B_III_	2
3*x*	3*x*	1*x*	4*x*	9*x* ^a^	1:0^b^	B_III_	1
3*x*	3*x*	1*x*	4*x*	10*x* ^a^	9:1	B_III_	1
							(Total 6)
Tetraploid							
4*x*	2*x*	0*x*	2*x*	6*x*	2:1	Polyhaploid	12
4*x*	2*x*	1*x*	3*x*	5*x*	4:1	Irregular male meiosis	2
4*x*	2*x*	1*x*	3*x*	6*x* ^a/d^	1:0^b^	”	1
4*x*	2*x*	1*x*	3*x*	7*x* ^a^	3:1	”	4
4*x*	3*x* ^c^	2*x*	3*x*	8*x*	3:1	Irregular female meiosis	3
4*x*	4*x*	2*x*	6*x*	10*x*	4:1	B_III_	10
4*x*	4*x*	2*x*	6*x*	14*x* ^a^	6:1	B_III_	1
							(Total 33)

^a^ Potential trinucleate endosperm or mitotic nondisjunction in one of both polar nuclei

^b^ Autonomous endosperm

^c^ Aneuploidy

^d^ Fertilization by two sperm nuclei

***N*** number of seeds

### Triploid bridge and triploid block

Due to the availability of maternal leaf ploidy data for all plants, embryos with higher ploidy than the mother plant could be determined with high certainty. To investigate hypothetical presence of a triploid bridge in *R. kuepferi*, we assessed the occurrence of triploid embryos among diploid mother plants, as well as tetraploid embryos derived from triploid plants as potential evidence. Focusing on maternal-to-paternal genome ratios in endosperm, deviations from the normal 2:1 state were interpreted as potential triploid block. According to Harlan and de Wet (1975), tetraploidy principally can be reversible which is part of polyploid evolution. Tetraploids producing lower- or even higher-ploidy offspring may be detrimental for survival due to minor cytotype exclusion as a function of ploidy shift frequency. Hence, we also documented all cases of tetraploids that had produced embryos of divergent ploidies. Triploid progeny of tetraploids, if not based upon fertilized reduced egg cells, suffers from aneuploidy and chromosomal deficiencies that may enhance implications of a potential triploid block.

### Pollen size and stainability

Three-dimensional pollen size (or volume) was determined on a Multisizer 3 (Beckman Coulter, Brea, California, United States) following pollen preparation methods described by De Storme et al. (2012) with minor modifications. From 179 *R. kuepferi* plants, mature stamens from the outer rim of single flowers were collected, dried in silica gel and cut in half prior to soaking them for at least 15 min in 5 ml ISOTON II (Beckman Coulter, Brea, California, United States) in accuvette cups (Beckman Coulter, Brea, California, United States). To check for any significant influence of incubation time on pollen size, we conducted time series analyses of randomly chosen pollen samples (3 di- and 4 tetraploid) measuring same samples 9 times, with 15 min time between the distinct measurements (120 min). These test series revealed no significant change of mean pollen diameter (*P* = 0.59) and pollen size distribution (*P* = 0.43) among samples (Online Resource 1), indicating that incubation time does not influence the pollen size. At least 10,000 particles per sample were counted of which approximately 2,000–3,000 were within the estimated pollen size range. Histogram peaks of pollen diameters were generated by and analyzed with the Multisizer 3 Control Software 3.53 (Beckman Coulter, Brea, California, United States).

For microscopy, mature pollen grains were stained with a 10% acetic orcein solution for 15 min and viewed with a Zeiss Apotome 2 microscope (Carl Zeiss AG, Oberkochen, Germany) at 400 × magnification. At least ten stamens per plant were analyzed, and diameters of 80–120 pollen grains per sample (depending on quality and quantity of available anthers) were measured with ZEN operating software (Carl Zeiss AG, Oberkochen, Germany). To determine the different size classes of viable pollen, we further carried out a stainability test, using a 10% I_2_-KI solution (Lugol’s iodine) for detection of starch content as an indicator for mature viable pollen (Wang et al. 2004). A light microscope (Leica DM5500B with DFC 450 C camera, LAS V41 Software, Leica Microsystems, Wetzlar, Germany) at a 400x magnification was used to discriminate black-stained, viable pollen from brownish, reddish and translucent (empty) pollen which were all considered as non-viable (Stebbins 1950; Tie et al. 2006).

### Statistical analyses

Independent *T* and *F* tests were performed to check for significant differences in pollen size and respective proportions among size classes between diploids and tetraploids. All calculations were executed in R version 3.1.2 (R Core Team 2014). Prior to analysis, percentages were arcsine-transformed to improve normal distribution of the data.

## Results

### Flow cytometric seed screening

**Fig. 2:**
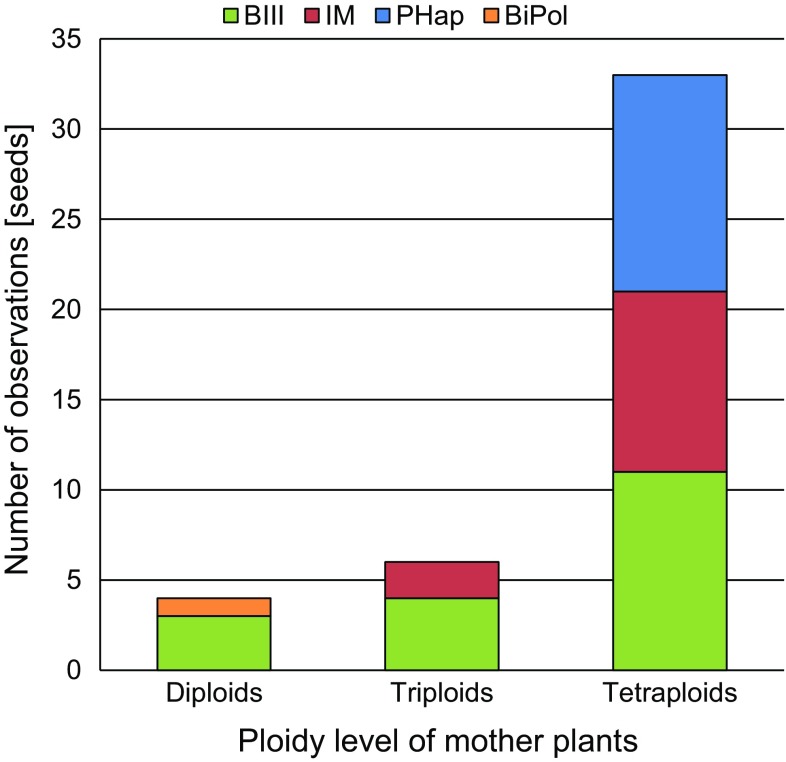
Barplot of seed formation pathways among the 43 seeds with ploidy shifts in the embryo inferred from FCSS in *Ranunculus kuepferi* (see Table 1). Pathways were grouped in four different classes based on main reproduction mode. *BIII* B_III_ hybrids; *IM* irregular meiosis; *PHap* polyhaploids; *BiPol* biparental polyploidization

The ploidy level of 551 individual mother plants (132 diploid, 25 triploid and 394 tetraploid) and the embryo/endosperm ploidy pattern in 2795 seeds were determined. The great majority of seeds had no ploidy shift in the embryo compared to the mother plant and was either formed from a sexual or a fully apomictic pathway, resulting in diploid and tetraploid embryos, respectively (see details and representative flow histograms in Schinkel et al. 2016). We present here 43 seeds (1.5% of all seeds) showing a shift in the ploidy level of the embryo compared to the mother plant (see Online Resource 2). Four of these seeds occurred in diploid, six in triploid and 33 in tetraploid mother plants (Table 1 and Fig. 2). Three seeds of diploid mother plants contained a triploid (3*x*) embryo accompanied by a pentaploid (5*x*) endosperm (B_III_ hybrids). The observed ratio indicates for the involvement of unreduced female gametes, resulting in a diploid egg cell (2*Cx(m)*) fertilized by one haploid sperm nucleus of a reduced pollen (1*Cx(p)*), as well as two fused diploid polar nuclei (2*Cx(m)* + 2*Cx(m)* = 4*Cx(m)*) fertilized by the second haploid sperm nucleus (1*Cx(p)*).

The fourth seed contained a tetraploid (4*x*) embryo together with a hexaploid (6*x*) endosperm, indicating the additional involvement of an unreduced pollen grain. Here, the endosperm had a ratio of 4C*x*(*m*):2C*x*(*p*), resembling the optimal maternal–paternal allocation of 2:1 (Lin 1984). Strikingly, no triploid resulting from the fusion of an unreduced pollen with a reduced embryo sac could be found, as this would show an embryo/endosperm ratio of 3:4 (1*Cx(m)* + 2*Cx(p)* in the embryo, and 2C*x*(*m*) + 2C*x*(*p*) in the endosperm.

Of the six cases of ploidy shifts of the embryo in triploid mother plants (found within 125 analyzed seeds), four seeds contained tetraploid (4*x*) embryos. Another two seeds contained diploid (2*x*) embryos, both with triploid (3*x*) endosperm indicating double fertilization by reduced pollen of either a diploid or a likewise triploid donor (euploid 1*x* pollen).

For the 33 seeds with embryo ploidy shifts from their tetraploid mothers, the resulting combinations of embryo and endosperm ploidy and the associated developmental pathways were rather complex (see Table 1). We found 12 seeds exhibiting diploid embryos with hexaploid endosperm, likely a consequence of polyhaploid embryo formation (reduced, unfertilized egg cell, the polar nuclei fertilized with two reduced sperm nuclei). Ten triploid embryos with endosperm ploidies ranging from penta- to octaploid can be explained by irregular female or male meiosis as observed previously by Cosendai and Hörandl (2010). Eleven seeds had hexaploid embryos with either deca- or tetradecaploid endosperm, representing B_III_ hybrids with unreduced female gamete formation. Explanations of some higher-ploidy endosperm in tetra- (6*x*, 7*x* for diploid embryos, 14*x* for hexaploid embryos) as well as triploids (9*x*, 10*x*) remain elusive, although according to Talent and Dickinson (2007) trinucleate endosperm could explain some of the observed patterns.

### Pollen analysis

**Fig. 3:**
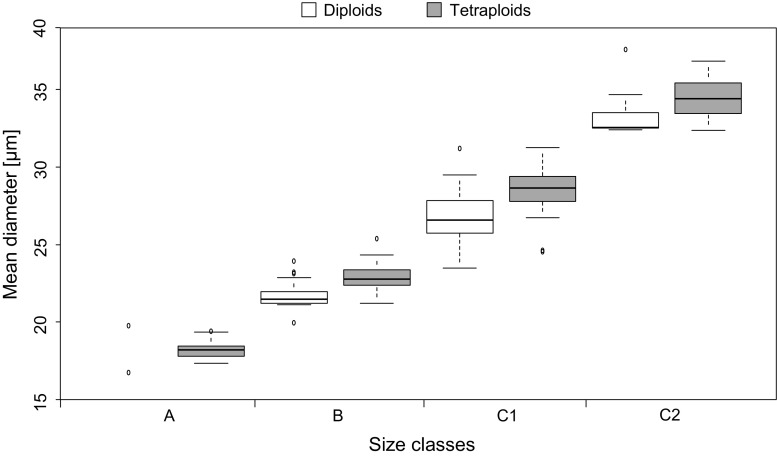
Boxplot of volumetric pollen measurements in *Ranunculus kuepferi* (see Table 2). Results were calculated as diameters and grouped in four size classes. A: smallest, with diameters under 19 µm; B: small, 19–27 µm; C1: large, 27–33 µm; C2: largest, with diameters above 33 µm

**Fig. 4:**
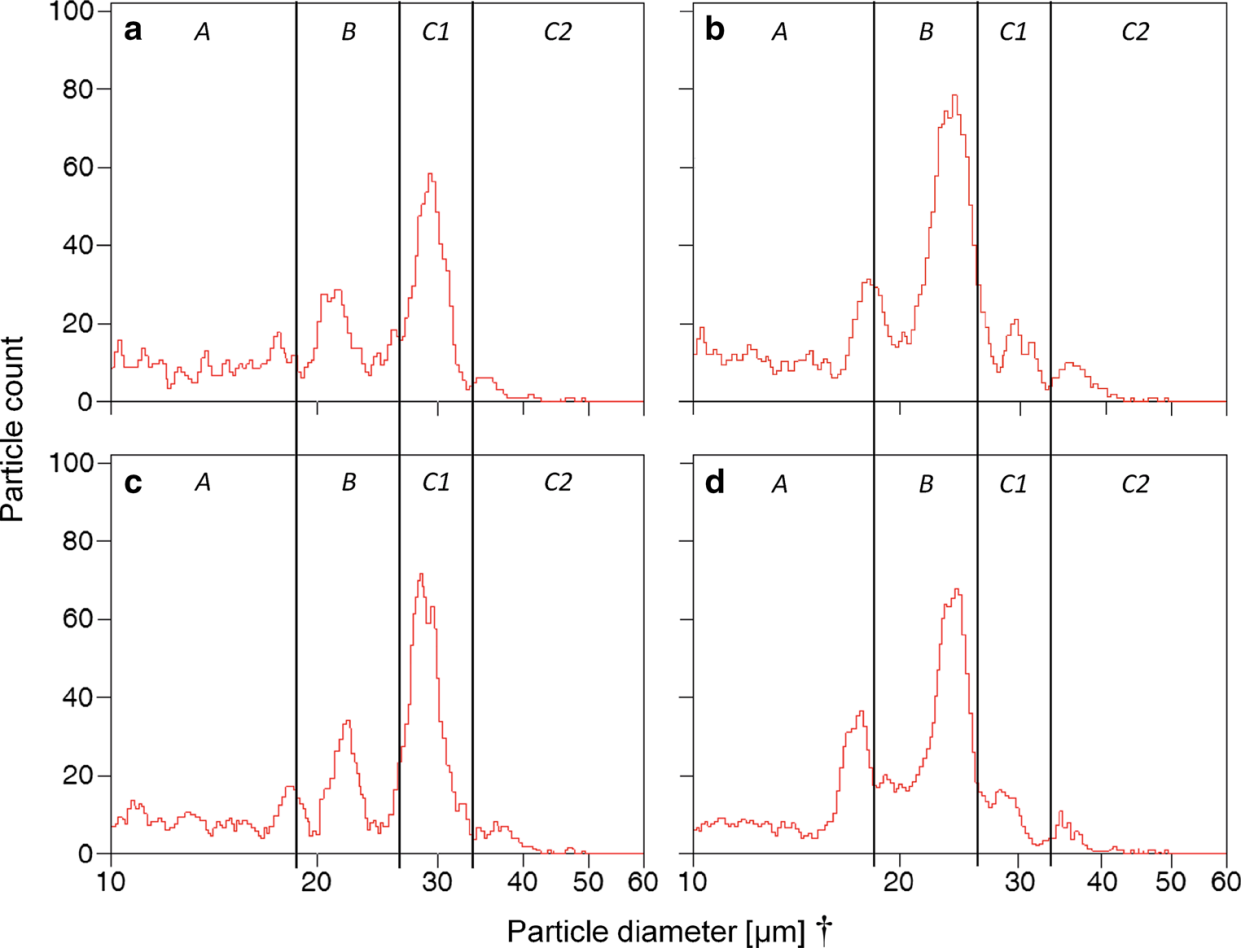
Histograms of size classes of volumetric pollen measurements of two diploid (**a**, **c**) and two tetraploid (**b**, **d**) individuals. *Vertical black lines* show margins of size class ranges. *PK* peak; ^†^ particle diameter was automatically calculated in Multisizer 3 Control Software 3.53 (Beckman Coulter, Brea, California, United States) based on volumetric measures

**Fig. 5:**
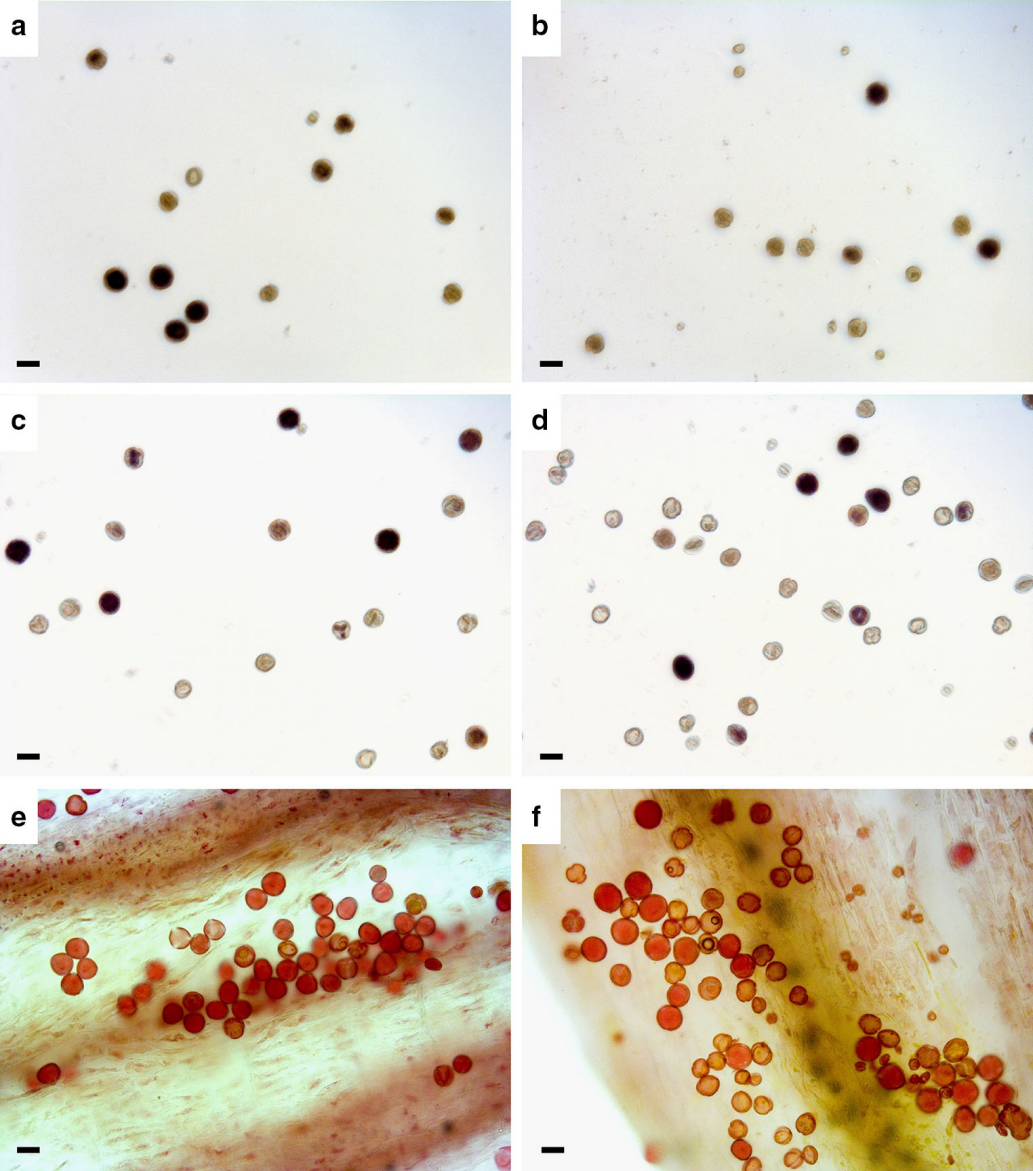
Microscopic pictures showing pollen of *Ranunculus kuepferi* representing different size classes. Pollen was stained with either 10% I_2_-KI solution (Lugol’s iodine) (viability staining;** a**–**d**) or 10% acetic orcein (manual size measurement; **e**–**f**). Pollen from three diploid (**a**, **c**, **e**) and three tetraploid (**b**, **d**, **f**) plants are shown. For viability assessment with I_2_-KI, pollen grains stained black were judged as viable, and those staining *brownish* to *light yellowish* were considered sterile (**a**–**d**). Acetic orcein pictures were taken directly from anthers and serve as visual illustration. Bar corresponds to 27 µm (approximate delimitation between mostly non-viable [<27 µm] and mostly viable [>27 µm] pollen)

Pollen size measurements revealed a large spectrum of diameters in both tetraploid and diploid progenitor plants. Ranges in both were very similar, varying from 15.8–39.7 µm in tetraploids and 15.6–38.3 µm in diploids (Fig. 3). Histograms of most samples peaked at approximately the same four sizes (Fig. 4). Therefore, we assorted observed counts obtained from the histograms into four size classes (Table 2) of very small (A), small (B), larger (C1) and very large (C2) pollen. The classification is based on our microscopic observations, which revealed pollen grains of varying sizes and abundance reasonably consistent with the volumetric measurements (Fig. 5). Particles smaller than 19 µm were either strongly deformed, were deeply grooved pollen or represented immature pollen in development as well as cell debris from preparation. Particles in the range from 19 to 27 µm appeared to be empty pollen seemingly stuck in development. Only particles greater than 27 µm appeared to be fully developed pollen, showing common characteristics of viable tricolpate–psilate pollen as typical for many *Ranunculus* species (Huber 1988; Izmailow 1996; Hörandl et al. 1997). Everything above 33 µm diameter looked like bloated pollen, sometimes exhibiting signs of disintegration, or was debris (e.g., larger tissue segments) in the solution. We did not observe pollen with incomplete tetrade disintegration or any form of other random pollen aggregation.

**Table 2:** Deduced size classes, average sizes and proportions from pollen volumetric measurements

	Beckman Coulter Multisizer 3				Microscope					
	*x* ≤ 19	19 < *x* ≤ 27	27 < *x* ≤ 33	*x* > 33	Aborted	Non-viable	Viable, small	Viable, large	Viable	Non-viable
	A	B	C1	C2	A	B	C1	C2		
Diploids										
*N*	15,184	53,841	112,859	9101	126	1925	3040	1416	2868	3639
Ø*N*/individual	287	1146	1710	1011	2	29	45	21	43	54
Mean diameter [µm]	18.3 ± 0.6	20.6 ± 4.7	26.7 ± 1.5	33.4 ± 1.7						
Proportion [%]	8.0	28.2	59.1	4.8	1.9	29.6	46.7	21.8	44.1	55.9
Tetraploids										
*N*	24,893	120,724	69,693	12,266	1463	4439	2396	1112	2892	6518
Ø*N*/individual	372	1548	882	371	16	49	27	12	32	72
Mean diameter [µm]	18.2 ± 0.4	22.6 ± 2.4	28.6 ± 1.2	34.4 ± 1.3						
Proportion [%]	10.9	53.0	30.6	5.4	15.5	47.2	25.5	11.8	30.7	69.3

Sizes correspond to diameters; size means and proportions are calculated with pooled results of all measured pollen per cytotype

*N* total number of measurements (particles), *ØN/individual* mean number of measurements (particles) per individual

Generally, average diameters of pollen isolated from diploids were smaller than those of tetraploids except for diameters in size class A, wherein counted particles were slightly but not significantly bigger in diploids [*t*(89) = 0.1, *P* = 0.95]. Observed differences were highly significant in size classes B [*t*(89) = −7.2, *P* < 0.01] and C1 [*t*(89) = −3.3, *P* < 0.01] (Table 2), whereas differences between the pollen from di- and tetraploids in class C2 were only marginally significant [*t*(89) = −2.2, *P* = 0.04].

No significant difference in diploid/tetraploid proportions was found in class A [*F*(151) = 2.0, *P* = 0.16] (Fig. 3). Classes B and C1 differed significantly with tetraploids comprising more of the smaller (B; [*F*(151) = 16.7, *P* < 0.01]) and diploids more of the larger (C1; [*F*(151) = 29.3, *P* < 0.01]) pollen. Very large pollen of size class C2 were significantly more frequent in tetraploids [*F*(151) = 5.5, *P* = 0.02] compared to diploids.

Stainability analysis with Lugol’s iodine revealed tetraploids to have significantly less viable pollen (mean of 30.7%) compared to diploids (mean 44.1%) [*t*(66) = −4.76, *P* < 0.01]. Interestingly, in both cytotypes pollen with diameters smaller than 27 µm was not well developed. Nearly all viable pollen observed during microscopy possessed diameters greater than 27 µm and was therefore assigned to size classes C1 and C2.

## Discussion

We here report neopolyploid formation via unreduced eggs in wild populations of the alpine plant *Ranunculus kuepferi* and call this pathway of polyploidization a female triploid bridge. Additional to the overall ploidy distribution and reproduction pathways that maintain the ploidy level (Schinkel et al. 2016), we revealed the events that lead to polyploid formation based on ploidy shifts in the seeds (embryos and endosperm) of di-, tri- and tetraploid mother plants.

### Developmental pathways

Among diploid progenitors, we identified three of four seeds with triploid (3*x*) embryos and pentaploid (5*x*) endosperm derived from two fused diploid (2*x*) nuclei of the central cell fertilized by a reduced pollen sperm nucleus (1*x*). Hence, the unreduced egg cell and central cell have been fertilized each by the two haploid (1*x*) sperm derived from a reduced pollen. Ramsey and Schemske (1998) found a similar pattern in a comparison of 4*x* × 2*x* versus 2*x* × 4*x* crosses.

Although these findings indicate that triploid bridge-mediated polyploidy induction in diploid *R. kuepferi* occurs through the formation of unreduced female gametes, most literature on restitutional meiosis assesses rates of unreduced 2*n* pollen and neglects the process of female gametogenesis (Mirzaghaderi and Hörandl 2016). Flow cytometric seed screening informs about male vs. female unreduced gamete formation because embryo/endosperm ploidies are different. When a normal meiotically reduced haploid egg cell of a diploid mother plant is fertilized by an unreduced 2*n* pollen, the resulting embryo would be triploid (3*x*) accompanied by an tetraploid (4*x*) endosperm possessing a 2:2 maternal-to-paternal genome balance (2C*x*(*m*) from the two polar nuclei, 2C*x*(*p*) from the second sperm nucleus). A key finding of our studies is that we did not find any case of such triploid seed, which indicates that no male triploidization by unreduced 2*n* pollen took place in our sample. Moreover, these results also reveal that it is highly unlikely that triploids in the sympatric geographical zone originated from backcrossing of tetraploid pollen donors to diploid mother plants (see map in Schinkel et al. 2016). Tetraploids typically produce reduced pollen with a diploid chromosome set (2*x*). Events whereby diploid pollen fertilize the egg of a sexual diploid would also result in triploid embryos accompanied by tetraploid endosperm (3:4). However, we did not find a single seed with this ploidy structure. Hence, we cannot confirm the theoretical possibility of a secondary origin of triploids, which is further impeded by low fertility (Hörandl and Temsch 2009).

However, we also found evidence for 2*n* pollen being involved in biparental polyploidization in *R. kuepferi*. One seed among diploids revealed a tetraploid (4*x*) embryo and a hexaploid (6*x*) endosperm, which can only result from an unreduced diploid (2*x*) pollen that has fertilized both the egg (2*x*) and central cell (4*x*) of an unreduced (2*x*) embryo sac. Due to the involvement of both unreduced male and female gametes, the mechanism of autotetraploidization here would be a biparental process rather than a uniparental one, as would be the case if the polyploidization was entirely based on either unreduced female or male gametes. However, such biparental polyploidization may be also caused by reduced pollen of surrounding higher-ploidy individuals, including aneuploid pollen of triploids. This case is more likely as the seed was formed in a population of mixed ploidies in the contact zone of cytotypes (Schinkel et al. 2016).

It is generally thought that triploids have low fertility or even are totally sterile. Indeed, in many species, difficulties in the production of viable seeds and pollen restrict their role in tetraploid formation (Ramsey and Schemske 1998). However, recent studies that estimated fertility of triploids suggested that triploids in many taxa are able to produce some euploid gametes and are therefore often semi-fertile (Ramsey and Schemske 1998). Accordingly, in our previous study on *R. kuepferi* (Schinkel et al. 2016) we had obtained 125 fully developed seeds from 25 triploid plants, which we analyzed with DNA flow cytometry. Most seeds consisted of a triploid embryo and a pentaploid endosperm, when produced sexually (six seeds), or hepta- as well as octaploid endosperm, when produced apomictically (119 seeds). Although seed set in triploids is lowest among the three analyzed cytotypes (Schinkel et al. 2016), these findings suggests that triploids are indeed fertile to some degree, not least because they mainly reproduce via apomixis. Switching the reproductive path to apomixis is a common strategy of polyploid plants to escape sexual-based F1sterility (e.g., Hojsgaard et al. 2014) and thus constitutes an important factor in the establishment of a persistent amount of triploids among diploid populations, which is sufficient to allow further polyploidization steps.

Focusing on the six seeds of triploid individuals that featured a ploidy shift of the embryo, four seeds possessed tetraploid (4*x*) embryos. Heptaploid (7*x*) endosperm of two of these seeds indicates an origin from unreduced egg cells fertilized by euploid reduced 1*x* pollen of either a triploid or diploid donor. Such a pathway can be described as unilateral female sexual tetraploidization involving unreduced (3*x*) embryo sacs. The remaining two seeds with tetraploid embryos were accompanied by a nonaploid or a decaploid endosperm, respectively. In both cases, the most plausible explanation for the observed patterns is the presence of trinucleate endosperm (Talent and Dickinson 2007), since unreduced triploid pollen can explain nonaploid endosperm, but would cause hexaploid embryos. Certainly, trinucleate nonaploid endosperm implies that the endosperm developed autonomously, i.e., without fertilization (pseudogamy), as we previously found in some seeds of tetraploid individuals (Schinkel et al. 2016). In the case of decaploid endosperm, we assume a normal double fertilization of the unreduced egg cell and the trinucleate endosperm by a euploid reduced 1*x* pollen (diploid/triploid donor). Besides the four seeds comprising tetraploid embryos, two seeds were detected with diploid embryos and a triploid endosperm. Although comprising the same embryo/endosperm condition as regularly formed seeds of sexual diploids, it remains elusive whether these seeds would be fertile or not.

In comparison with triploids, an even larger variety in possible mating outcomes was observed in the progeny of tetraploid mother plants. Besides the most common, sexual and asexual reproduction without changes of embryo ploidy (Schinkel et al. 2016), we found 33 seeds in which a ploidy shift in the embryo could be detected. Interestingly, most of these seeds (12) contained a polyhaploid embryo, i.e., resulting from a reduced, unfertilized egg cell (Table 1). The other seeds contained either again B_III_ offspring (unreduced female gametes fertilized by reduced male gametes) or reduced, but triploid embryos and 6*x* to 8*x* endosperm (Table 1). This last developmental pathway can only be interpreted with an irregular female meiosis resulting in aneuploid gametes. Such seeds were also found regularly in a previous study within tetraploid populations outside the contact zone of diploids and tetraploids (Cosendai and Hörandl 2010). Their formation may be explained by multivalent formation and unequal chromosome segregation during meiosis in these autotetraploid plants, resulting in aneuploid megaspores (see Cosendai et al. 2011).

### Endosperm imbalance and triploid block

As in other plants, triploids of *R. kuepferi* have a lower seed set than diploids and tetraploids (Schinkel et al. 2016). Seed quality is in most angiosperms strongly dependent on proper endosperm formation as it is the nutritious tissue for the embryo. The ratio of two maternal-to-paternal genome copies appears to be optimal (Scott 2007; Köhler et al. 2010). Paternal genes proliferate endosperm growth, while maternal genes downregulate growth but are important for normal cellularization of endosperm (Spielmann et al. 2003). Genomic imprinting, i.e., the differential expression of paternally and maternally inherited genes, is another potential factor of endosperm imbalance (Haig and Westoby 1991; Vinkenoog et al. 2003; Köhler et al. 2010). Interploidy crosses affect the endosperm balance by altering maternal-to-paternal genome contributions. However, some pseudogamous apomicts developed mechanisms to overcome detrimental effects resulting from such deviations, for example conformation in megagametophyte structure and/or fertilization behavior, in particular dispermy (sexual polyploidization), as well as endosperm fertilization under inclusion of both sperm nuclei (Talent and Dickinson 2007; Šarhanová et al. 2012; Ludwig et al. 2013; Burgess et al. 2014). While the female triploid bridge via unreduced egg cells and polar nuclei will result in triploid embryos accompanied by an unbalanced endosperm containing a maternal-to-paternal genome ratio of 4*m*:1*p*, the male triploid bridge would result in a maternal-to-paternal genome ratio of 2*m*:2*p* in the endosperm. In *R. kuepferi*, we only observed the pathway of unreduced female gamete formation, which may have less severe effects on endosperm (smaller, but cellularized) than the paternal excess (over-proliferated, but not cellularized; see Spielmann et al. 2003 on *Arabidopsis*). Observations of rare cases of autonomous endosperm formation in Cosendai and Hörandl (2010) and Schinkel et al. (2016) support the hypothesis that the species *R. kuepferi* tolerates a lower paternal genome contribution to endosperm better than a paternal excess. Hence, these findings together with data presented in this study demonstrate the triploid block in *R. kuepferi* is not strict and still allows for triploid seed formation at low frequencies.

### Pollen ploidy

In many plant taxa, pollen ploidy can directly be assessed by pollen size measurements (Ramsey and Schemske 1998). Hence, differences in diameter of reduced and unreduced pollen should be likewise detectable. Several studies reported that the diameter of 2*n* pollen grains is often 30–40% larger than that of reduced pollen and that size distribution of reduced and unreduced pollen is mostly bimodal (e.g., Róis et al. 2012; Cohen et al. 2013; De Storme et al. 2013; Marinho et al. 2014; Rotreklová and Krahulcová 2016). Huber (1988) reported significant differences in diameter between pollen of di- and tetraploids in *R. kuepferi*, with diploid pollen being on average smaller than tetraploid pollen. Our microscopic investigations and results of a high-throughput pollen sizing method, measuring pollen diameters of several thousand pollen grains per individual volumetrically, in fact revealed a greater variation in size and shape than previously thought. Pollen of both cytotypes consisted of grains assignable to four distinctive size classes, found in pollen samples of every single individual. In accordance with the results of Huber (1988), most pollen grains in tetraploids have a slightly larger mean diameter (size classes B, C1 and C2; see Table 2), when compared to pollen grains isolated from diploid plants in the same size ranges. Discernible and significant differences between pollen from di- and tetraploids occur mainly in proportions of observed pollen in one size class or another. Considering the differences in the distribution of the counted pollen among the different size classes as a whole, it is particularly striking that most pollen grains of tetraploids (53.0%) were found in the smaller size class B (see Table 2, Fig. 3). Our microscopic studies generally confirmed these findings and additionally identified the smaller size classes as non-viable. Shriveled, misshapen and very small pollen grains (size class A), as well as normally shaped pollen grains with a diameter under 27 µm (size class B), were not stainable, which accounts for more than two-third of all measured pollen in tetraploids. The unusual round and smooth shape of very large pollen (size class C2) suggests that these might be as well infertile even though they contain high levels of starch, as shown by Lugol’s iodine staining. Likely, only pollen grains with a diameter in the size range between 27 and 33 µm (size class C1) seem to represent fully developed, mature, viable pollen that can contribute to successful fertilization. A huge amount of aborted pollen grains and a highly variable pollen size as found in *R. kuepferi* is typical for facultatively apomictic plants (Izmailow 1996; Hörandl et al. 1997; Voigt et al. 2007) and likely results from meiotic and developmental disturbances during male gametophyte formation (e.g., Hojsgaard et al. 2014).

The observed differences in the proportions of size classes between the volumetric measurements of the Multisizer 3 and those detected by microscopy might be explained by the much larger number of pollen counts within samples that were automatically measured and/or by biases through subjective preference of measuring well-formed pollen during direct visual observation. Very big and especially very small particles further contribute to the fractions of size class A and C2, where highest deviations between measurements were found. Hence, the high-throughput method of volume-based particle size measurements allows for an accurate quantification of pollen size analysis, but is more informative if combined with the time-consuming method of direct optical examination by microscope, at least in our model system.

Because of the heterogeneity of pollen size and the overlap of ranges in diameter of viable pollen between diploid and tetraploids, we cannot directly infer pollen ploidy from pollen size in *R. kuepferi*. Hence, the assessment of actual frequencies and effects of unreduced and aneuploid male gamete formation still needs further investigation. Considering our flow cytometric seed screening results compared to our volumetric and microscopic pollen data in conjunction with the constraints of endosperm balance discussed above, we conclude that unreduced pollen are most likely only involved in rare cases of biparental sexual polyploidization, i.e., when the female gametes are unreduced too.

### Mechanisms of unreduced gamete formation

Although we observed female and male gamete formations only indirectly, some tentative conclusions can be drawn on the mechanisms of meiotic non-reduction. We suppose that apospory rather than restitutional meiosis is the major pathway to polyploidization in *R. kuepferi*. The great variation of pollen size classes and the regular occurrence of aneuploidy in female gametophytes of tetraploid plants (Table 1) suggest that unbalanced meiosis happens frequently in both male and female developments. The resulting micro- and megaspores have various unbalanced chromosome complements. During apomictic female gametophyte development, unreduced embryo sac formation happens in *R. kuepferi* via apospory (Burnier et al. 2009), which is also well documented for other *Ranunculus* species (Nogler 1984; Hojsgaard et al. 2014). Diplospory, as a result of female restitutional meiosis, has never been reported for *Ranunculus*. In aposporous 2*n* megaspore formation, a somatic cell of the nucellus takes over gametophyte identity and development, while meiotic products abort. This way, female gametophyte development bypasses all detrimental effects of disturbed chromosomal segregation at meiosis and instead produces megaspores with complete unreduced chromosome sets. For male reproductive development, no alternative to meiosis is available, and reduced, unreduced and unbalanced pollen is probably produced in parallel. Selection on euploid male gametes and constraints of endosperm balance will probably favor the involvement of normally reduced pollen grains (i.e., haploid) for fertilization and final seed development, while unreduced pollen appear to be rarely successful.

### Implications for the evolution of polyploidy and apomixis

**Fig. 6:**
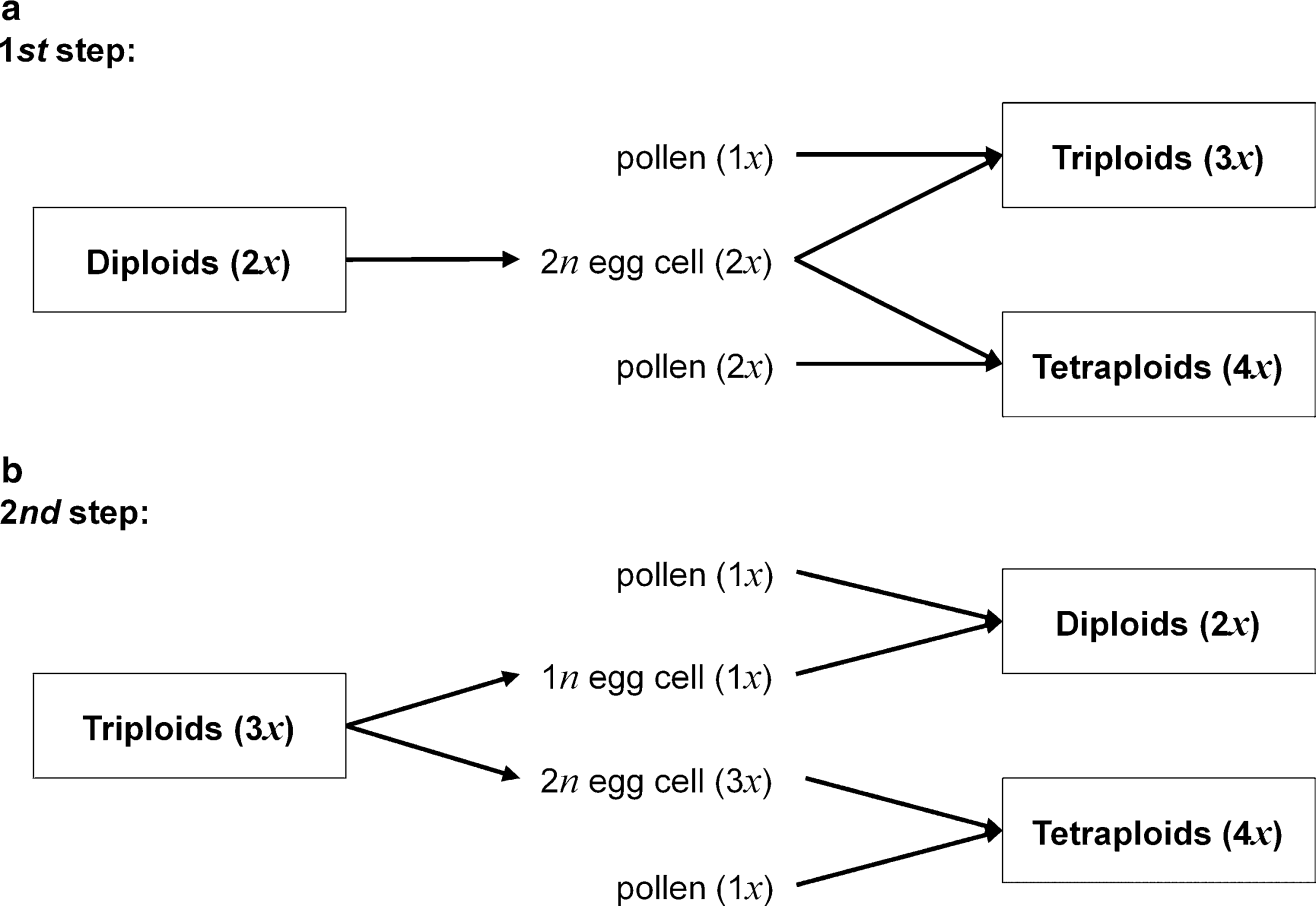
Scheme representing different observed pathways of evolutionary origin of polyploids among *Ranunculus kuepferi*. Triploid formation is depicted as first step of a female triploid bridge in **a**, tetraploid formation as second step in **b**

Our data suggest that polyploidization in *R. kuepferi* mainly occurs through female triploid bridge, as unreduced female gamete formation was observed in all cytotypes. This way, triploids can easily be generated, and in turn produce tetraploid offspring (Fig. 6). The pathway of a male triploid bridge was not observed in this species. Since the capability to form unreduced female gametophytes via apospory is supposed to be a heritable trait (Nogler 1984; Ozias-Akins and Van Dijk 2007), tetraploids that originated from the described female variant of unilateral sexual polyploidization should be able to inherit this trait to the offspring. Continued fertilization and B_III_ offspring formation would result in constant increase in ploidy levels, which is, however, limited by cellular constraints (Comai 2005). In contrast, if regular unreduced embryo sac formation is coupled to parthenogenetic development, then the plants can keep ploidy levels constant in the offspring and reproduce fully asexually via seeds (apomixis). Such a fully functional apomixis is the predominant mode of reproduction in tetraploid *R. kuepferi* populations (Schinkel et al. 2016). Strikingly, fully functional apomixis (with unreduced, unfertilized egg cells) has already been observed in some diploid populations (Schinkel et al. 2016), further confirming a general capacity and genetic determination for unreduced female gamete formation. Among tetraploids, these previous studies on *R. kuepferi* did not detect a single tetraploid individual that shows full sexual reproduction, which would be expected if polyploidization is caused by unreduced pollen as unilateral, sexual and male-dependent process. Based on the absence of 3*x* seeds resulting from male 2*n* gametes, we thus assume that sexual tetraploid populations probably never originated in *R. kuepferi*.

The form of unreduced egg cell formation via apospory not only facilitates polyploidization but additionally aids in the establishment of the apomictic mode of reproduction. The conclusion that partial apomixis is rather cause than consequence of polyploidy contradicts older theories which postulated polyploidy to be a precondition for expression of apomixis (Nogler 1984; Carman 1997; Koltunow and Grossniklaus 2003). According to our results, it is rather the other way round. First, apospory appears in diploids, resulting in polyploidization. Then, after coupling to parthenogenesis, apomixis would be established in the polyploid line. These findings also support the idea of apomixis being a springboard for polyploidization and further evolution rather than a dead end (Hörandl and Hojsgaard 2012). The coupling of unreduced egg cell formation to parthenogenesis has further implications for the establishment of newly formed tetraploids. Sexual neopolyploids often suffer from a minority cytotype disadvantage (Levin 1975), as they emerge as single or a few individuals in a predominantly diploid population, where they would be fertilized predominantly with “wrong” haploid pollen. Triploid embryo formation and the above-discussed endosperm imbalances would lower their fitness and impede establishment of polyploids. In contrast, the shift to apomixis and self-fertility can overcome this problem and allow for establishment of polyploid populations even via a single or a few tetraploid individuals. This advantage of tetraploid apomictic populations, among others, may have contributed to the observed large distributions of tetraploid *R. kuepferi*, in contrast to sexual diploid populations which failed to expand their geographical area after the last glaciation (Schinkel et al. 2016; Kirchheimer et al. 2016).

## Information on Supplementary Material

Online Resource 1 Diagram of time series analyses of pollen diameter measures after different incubation times on randomly chosen pollen samples.

Online Resource 2 Table of flow cytometry data of all individuals with peak position on flow histograms and total number of seeds.

### Electronic supplementary material

Below is the link to the electronic supplementary material.

Diagram of time series analyses of pollen diameter measures after different incubation times on randomly chosen pollen samples (DOCX 19 kb)

Table of flow cytometry data of all individuals with peak position on flow histograms and total number of seeds (DOCX 25 kb)
