# Supplementary material for: Pathways to polyploidy: indications of a female triploid bridge in the alpine species Ranunculus kuepferi (Ranunculaceae)
Source: Plant Syst Evol. 2017 Jul 6;303(8):1093–108. doi: 10.1007/s00606-017-1435-6 (PMC5640749; doi:10.1007/s00606-017-1435-6)
Supplement: Supplementary file 1 — Diagram of time series analyses of pollen diameter measures after different incubation times on randomly chosen pollen samples (DOCX 19 kb) [file 606_2017_1435_MOESM1_ESM.docx]

Plant Systematics and Evolution

*Special issue: Polyploidy in shallow and deep evolutionary times*

**Pathways to polyploidy: indications of a female triploid bridge in the alpine species *Ranunculus kuepferi* (Ranunculaceae)**

Christoph C. F. Schinkel^1^, Bernhard Kirchheimer^2^, Stefan Dullinger^2^, Danny Geelen^3^, Nico De Storme^3^, Elvira Hörandl^1*^

^1^Department of Systematics, Biodiversity and Evolution of Plants (with Herbarium), University of Goettingen, Untere Karspüle 2, 37073 Göttingen, Germany

^2^Department of Botany and Biodiversity Research, University of Vienna, Rennweg 14, 1030 Vienna, Austria

^3^Department of Plant Production, Faculty of Bioscience Engineering, Coupure Links 653, 9000 Gent, Belgium

^*^corresponding author: elvira.hoerandl@biologie.uni-goettingen.de

**Online Resource 1 – Time series of volumetric pollen diameter measures after different incubation times of ISOTON II solution on randomly chosen pollen samples**
