# Supplementary material for: Pathways to polyploidy: indications of a female triploid bridge in the alpine species Ranunculus kuepferi (Ranunculaceae)
Source: Plant Syst Evol. 2017 Jul 6;303(8):1093–108. doi: 10.1007/s00606-017-1435-6 (PMC5640749; doi:10.1007/s00606-017-1435-6)
Supplement: Supplementary file 2 — Table of flow cytometry data of all individuals with peak position on flow histograms and total number of seeds (DOCX 25 kb) [file 606_2017_1435_MOESM2_ESM.docx]

Plant Systematics and Evolution

*Special issue: Polyploidy in shallow and deep evolutionary times*

**Pathways to polyploidy: indications of a female triploid bridge in the alpine species *Ranunculus kuepferi* (Ranunculaceae)**

Christoph C. F. Schinkel^1^, Bernhard Kirchheimer^2^, Stefan Dullinger^2^, Danny Geelen^3^, Nico De Storme^3^, Elvira Hörandl^1*^

^1^Department of Systematics, Biodiversity and Evolution of Plants (with Herbarium), University of Goettingen, Untere Karspüle 2, 37073 Göttingen, Germany

^2^Department of Botany and Biodiversity Research, University of Vienna, Rennweg 14, 1030 Vienna, Austria

^3^Department Plant Production, Faculty of Bioscience Engineering, Coupure Links 653, 9000 Gent, Belgium

^*^corresponding author: elvira.hoerandl@biologie.uni-goettingen.de

**Online Resource 2 – Flow cytometry data.**

|  |  | *Embryo* | | |  | *Endosperm* | | |
| --- | --- | --- | --- | --- | --- | --- | --- | --- |
| **Individual** | **Ploidy** | **Position** | **CV [%]** | ***N*** |  | **Position** | **CV [%]** | ***N*** |
| 31-3-2 | 2*x* | 155.8 | 3.8 | 127 |  | 251.8 | 4.4 | 1808 |
| 204-1-1 | 2*x* | 192.6 | 4.8 | 178 |  | 279.6 | 4.7 | 1073 |
| 204-1-1 | 2*x* | 146.9 | 5.5 | 232 |  | 242.0 | 6.5 | 692 |
| 204-1-2 | 2*x* | 152.0 | 4.9 | 155 |  | 239.8 | 6.4 | 1279 |
|  |  |  |  |  |  |  |  |  |
| 200-1-2 | 3*x* | 190.3 | 4.0 | 239 |  | 515.6 | 5.2 | 913 |
| 204-3-2 | 3*x* | 93.1 | 5.2 | 219 |  | 303.7 | 4.3 | 1349 |
| 204-3-2 | 3*x* | 185.2 | 4.2 | 201 |  | 457.4 | 6.8 | 1222 |
| 204-3-2 | 3*x* | 190.1 | 4.1 | 230 |  | 332.0 | 4.8 | 867 |
| 204-3-3 | 3*x* | 92.2 | 4.8 | 232 |  | 300.1 | 5.3 | 1497 |
| 205-2-1 | 3*x* | 195.2 | 5.2 | 226 |  | 340.4 | 5.5 | 978 |
|  |  |  |  |  |  |  |  |  |
| 36-4-2 | 4*x* | 292.7 | 4.5 | 164 |  | 485.9 | 4.1 | 523 |
| 37-3-2 | 4*x* | 301.4 | 5.5 | 213 |  | 493.5 | 4.0 | 770 |
| 36-1-2 | 4*x* | 93.1 | 5.6 | 204 |  | 283.3 | 4.0 | 1093 |
| 36-1-2 | 4*x* | 143.7 | 3.1 | 93 |  | 373.5 | 5.2 | 419 |
| 40-4-3 | 4*x* | 282.9 | 2.3 | 117 |  | 674.4 | 3.1 | 1166 |
| 36-1-2 | 4*x* | 285.6 | 3.8 | 181 |  | 464.6 | 3.6 | 592 |
| 36-1-1 | 4*x* | 298.1 | 3.2 | 126 |  | 485.8 | 4.0 | 310 |
| 40-4-2 | 4*x* | 306.6 | 2.6 | 86 |  | 500.8 | 4.2 | 1566 |
| 45-1-1 | 4*x* | 300.3 | 2.5 | 84 |  | 492.7 | 3.9 | 569 |
| 54-2-1 | 4*x* | 289.2 | 4.3 | 145 |  | 482.4 | 4.0 | 408 |
| 58-2-3 | 4*x* | 312.6 | 2.6 | 210 |  | 515.7 | 3.8 | 875 |
| 58-2-2 | 4*x* | 312.8 | 3.1 | 147 |  | 539.5 | 3.6 | 705 |
| 73-1-3 | 4*x* | 289.4 | 1.7 | 104 |  | 480.4 | 2.5 | 1128 |
| 111-1-2 | 4*x* | 106.2 | 2.8 | 203 |  | 306.4 | 2.5 | 827 |
| 200-2-3 | 4*x* | 93.1 | 7.3 | 347 |  | 278.3 | 5.7 | 954 |
| 200-2-3 | 4*x* | 93.8 | 7.1 | 416 |  | 276.6 | 4.9 | 674 |
| 200-2-3 | 4*x* | 94.9 | 6.7 | 416 |  | 278.9 | 5.7 | 560 |
| 200-2-2 | 4*x* | 95.9 | 5.9 | 374 |  | 283.8 | 4.8 | 1100 |
| 200-1-3 | 4*x* | 96.7 | 5.2 | 459 |  | 284.5 | 5.0 | 1300 |
| 200-2-2 | 4*x* | 96.9 | 6.7 | 338 |  | 289.2 | 4.0 | 1039 |
| 200-1-3 | 4*x* | 99.3 | 6.0 | 374 |  | 295.6 | 5.9 | 802 |
| 200-1-3 | 4*x* | 101.2 | 6.2 | 396 |  | 297.3 | 4.5 | 1073 |
| 204-2-3 | 4*x* | 100.6 | 6.2 | 236 |  | 247.4 | 6.2 | 1067 |
| 204-2-3 | 4*x* | 144.8 | 4.8 | 166 |  | 279.9 | 5.6 | 937 |
| 205-4-2 | 4*x* | 139.0 | 5.2 | 213 |  | 347.6 | 5.4 | 829 |
| 204-3-1 | 4*x* | 96.4 | 5.1 | 261 |  | 280.1 | 4.9 | 1381 |
| 204-3-1 | 4*x* | 149.1 | 4.6 | 174 |  | 387.9 | 5.0 | 831 |
| 204-3-1 | 4*x* | 152.1 | 4.0 | 194 |  | 394.4 | 4.4 | 1211 |
| 204-3-1 | 4*x* | 152.6 | 4.0 | 171 |  | 394.3 | 4.4 | 1126 |
| 205-4-1 | 4*x* | 147.2 | 4.9 | 205 |  | 335.5 | 6.6 | 863 |
| 205-4-1 | 4*x* | 149.0 | 5.2 | 1037 |  | 244.6 | 5.1 | 390 |
| 205-4-1 | 4*x* | 145.4 | 5.8 | 252 |  | 370.3 | 5.5 | 520 |
| 205-4-1 | 4*x* | 147.6 | 4.6 | 378 |  | 382.7 | 4.7 | 799 |
